# Experience Lived by Iranian Patients with Irritable Bowel Syndrome: Transitory Crisis and Liberation

**DOI:** 10.17533/udea.iee.v37n3e10

**Published:** 2019-10-23

**Authors:** Zinat Mohebbi, Farkhondeh Sharif, Hamid Peyrovi, Mahnaz Rakhshan, Mahvash Alizade Naini, Ladan Zarshenas

**Affiliations:** 1 Ph.D. Assistant Professor, School of Nursing and Midwifery, Shiraz University of Medical Sciences, Shiraz, Iran. Email: mohebbi04@yahoo.com Shiraz University of Medical Sciences Shiraz Iran mohebbi04@yahoo.com; 2 Ph.D. Professor, School of Nursing and Midwifery, Shiraz University of Medical Sciences, Shiraz, Iran. Email: fsharif@sums.ac.ir; corresponding author Shiraz University of Medical Sciences Shiraz Iran fsharif@sums.ac.ir; 3 Ph.D. Professor, Nursing Care Research Center, Iran University of Medical Sciences, Tehran, Iran Iran University of Medical Sciences Iran University of Medical Sciences Tehran Iran; 4 Ph.D. Associate Professor, School of Nursing and Midwifery, Shiraz University of Medical Sciences, Shiraz, Iran. Email: mzrakhshan@gmail.com Shiraz University of Medical Sciences Shiraz Iran mzrakhshan@gmail.com; 5 Gastroenterologist. Associate Professor, Department of Gastroenterology and Hepatology, Shiraz University of Medical Sciences, Shiraz, Iran Shiraz University of Medical Sciences Shiraz Iran; 6 Ph.D. Associate Professor. School of Nursing and Midwifery, Shiraz University of Medical Sciences, Shiraz, Iran Shiraz University of Medical Sciences Shiraz Iran

**Keywords:** irritable bowel síndrome, qualitative research, hermeneutics., síndrome del colon irritable, investigación cualitativa, hermenéutica., síndrome do intestino irritável, pesquisa qualitativa, hermenéutica.

## Abstract

**Objective.:**

Research on the nature of living with Irritable Bowel Syndrome.

**Methods.:**

Qualitative study of the hermeneutical phenomenology, which conducted in-depth semi-structured interviews with people trained on the irritable bowel syndrome. The sampling was intentional type and open questions were used to collect data. The Thematic Analysis Method by Van Manen was used.

**Results.:**

Two principal themes and five subthemes emerged in this research to determine the meaning of living with the irritable bowel syndrome: *Storm in corporality* (body with pain and affliction, tension and sequence of symptoms, and: distress during moments of life) and *Relief* (sense of liberation of the body symptoms, and moments with pleasure).

**Conclusion.:**

The experience lived by patients with irritable bowel syndrome is of a transitory crisis and liberation.

## Resumen

**Objetivo.:**

Conocer la experiencia de vivir con el Síndrome del Intestino Irritable.

**Métodos.:**

Estudio cualitativo de la fenomenología hermenéutica en el que se realizaron entrevistas semiestructuradas en profundidad a personas con diagnóstico de síndrome del intestino irritable. El muestreo fue de tipo intencional y se utilizaron preguntas abiertas para recopilar los datos. Se empleó el método de análisis temático de Van Mennen.

**Resultados.:**

Dos temas principales y cinco subtemas emergieron en la presente investigación para determinar el significado de vivir con este síndrome: *Tormenta en la corporalidad* (cuerpo con dolor y aflicción, tensión y secuencia de síntomas, y: angustia en los momentos de la vida) y *Alivio* (sensación de liberación de los síntomas corporales, y momentos con placer).

**Conclusión.:**

La experiencia vivida de pacientes con síndrome del intestino irritable es de una crisis transitoria y de liberación.

## Resumo

**Objetivo.:**

Investigar a natureza de viver com a Síndrome do Intestino Irritável.

**Métodos.:**

Estudo qualitativo da fenomenologia hermenêutica na qual se realizaram entrevistas semiestruturadas em profundidade a pessoas com diagnóstico de síndrome do intestino irritável. A amostragem foi de tipo intencional e se utilizaram perguntas abertas para recopilar os dados. Se empregou o método de análises temático de Van Mennen.

**Resultados.:**

Dois temas principais e cinco subtemas emergiram na presente investigação para determinar o significado de viver com a Síndrome do Intestino Irritável: *Tormenta na corporalidade* (corpo com dor e aflição, tensão e sequência de sintomas, e: angustia nos momentos da vida) e *Alívio* (sensação de liberação dos sintomas corporais, e momentos com prazer).

**Conclusão.:**

A experiência vivida de pacientes com síndrome do intestino irritável é de uma crises transitórias e liberação.

## Introduction

Irritable Bowel Syndrome (IBS) is the most prevalent chronic functional gastrointestinal disorder (FGID) which is diagnosed by the change in intestinal habits (Diarrhea or Constipation alone or both together) and the existence of symptoms like bloating (swelling) and abdominal pain.(1) The prevalence of this disorder is varied throughout the world according to the various diagnostic criteria. The highest rate between 9 to 22 percent occurs in Unites States of America and European countries and the lowest rate, i.e. 4.2 and 4.4 percent, occurs in India and Thailand, respectively.(2) The IBS prevalence in Tehran has been shown to be about 25 percent by “Rome II” criterion.(3) Its prevalence in adults over 35 years in Shiraz (Iran) has been 10.9 percent.(4) Studies have shown that 50 to 70 percent of patients with IBS who are in search for treatment suffer from psychiatric comorbidity.(5) Another study in Iran showed that the mean score of quality of life of patients with IBS and its components are lower compared with healthy people. In addition, their mean score of mental health is higher than that of the healthy people, and the physical symptoms, anxiety and depression among patients with IBS were also more severe.(6) The expenses resulting from this disorder have been estimated to be about 2.8 million Dollars in Iran.(7) Patients with IBS had the maximum drug usage in Iran and the period of their absence from work had been 2.26 days more compared with patients with other functional digestive disorders.(8) 

The quantitative studies with positivism paradigm regarding the recommendations of the lifestyle, medicinal and educational interventions; dietary modification; eating habits; and also psychological intervention for such patients have contributed to relief from the effect of some aspects of this disorder on the living of sufferers to some extent.(9,10) Since quantitative measurements cannot often possess thoughts, feelings and experiences of people, the study of all aspects of life is possible via qualitative evaluation. The qualitative research focusing on human communications, feelings, thoughts, and understanding of individuals help the researchers to discover behaviors, feelings, and their experiences. Phenomenology as a research method is a rigorous, critical and systematic way to approach unknown phenomena. Its purpose is to comprehend the lived experience of certain phenomena, searching for meaning units of it.(11) In order to recognize the nature and meaning of life of patients with IBS, a review on qualitative studies was carried out. Based on this review, the patients state that the syndrome has influenced their daily performance, thoughts, feelings, and behaviors.(12) The physical and psychological effects,(13) socio-psychological consequences(14) and the experiences of changes in dietary programs(15) were also described by patients. In another study, patients expressed the methods of their adaptation with this disorder.(16) Most studies surveyed the experiences of patients with IBS and Inflammatory Bowel Disease (that has the organic cause) together or investigated a particular aspect of the experience of patients with IBS. In a few studies on the experience of patients with IBS in total, grounded theory(17) or descriptive phenomenology(18) was used. None of the mentioned studies has been carried out by hermeneutic phenomenology method which is a combination of description and interpretation. Also, no qualitative study regarding IBS has been conducted in Iran and other developing countries. It seems that, considering the existence of cultural, social, religious and believability background in our country, the knowledge of nurses for effective, comprehensive and holistic care of such patients is not sufficient. As a result, researchers followed this study with hermeneutic phenomenology approach in order to determine what the meaning of living with IBS is so as to perceive and extract their lived experiences about daily living.

## Methods

The researcher selected educational clinics (Shahid Motahari and Shahid Faghihi) and Kowsar clinic of Shiraz University of Medical Sciences as the research setting. Considering the objectives of the study, the participants were selected by purposeful sampling method, description and interpretation of experience of patients from living with IBS and was continued till rich, deep, powerful and relative results were obtained. Participants in the research included 15 patients with IBS who agreed to take part in the study. The inclusion criteria were suffering from IBS for a period of one year as diagnosed by a gastroenterologist through Rome III criterion, adults over 20 years, speaking Persian easily, having rich experiences in this regard and making appropriate communication, being inclined to present their experience to the researcher, having no acute psychological and cognitive disease and lack of organic disease like Inflammatory Bowel Disease (IBD) including Crohnʼs and Ulcerative Colitis.

Semi-structured deep interviews and taking note in place were used to collect and produce data. Expressing the purpose, assuring the participants regarding the confidentiality of information in all stages of the research, and obtaining informed consent from the participants, we carried out the interviews according to the guide questions after recording their demographic information by the researcher. Examples of the main questions of the current study included: “What does living with IBS look like?”, “What is the meaning of living with IBS?”, “What comes to your mind when we say IBS?”. The interviews lasted between 40 to 90 minutes. The total conversation was recorded after obtaining the patients’ permission. The recorded information was listened several times exactly after carrying out the interview at the possible shortest time. Thereafter, the interviews were written down word by word and analyzed. 

During the process of collecting and recording data, the researcher has used the mentioned anecdote recorded by Navab and Hajibabaee(19) quoted from Van Mennen(20) in order to facilitate this process. In the current study, the Hermeneutic phenomenology with an emphasis on Van Mennen viewpoint and method was used to discover the experience of patients as to living with IBS. The academy of Van Mennen is a descriptive and interpretative academy. He believes that description and interpretation are inseparable from each other. In this study, the six Van Mennen methodological themes were used as the research structure, as follows: 1- Turning to the nature of the lived experience: in this stage, the researcher was interested in understanding the nature of living with IBS at the time of the clinical trial research; 2- Investigating lived experience: selecting the participants with lived experience with IBS as their integral part was performed at this stage. Participants in their descriptions showed the nature of living with IBS from their personal experiences, and these descriptions were recorded for later written transcription; 3- Reflecting on the essential themes which characterize the phenomenon: in this stage, using the thematic analysis with selective and holistic technique, there was an attempt to determine the main themes from descriptions of the patients from lived experience with IBS. Also, after reflecting on each transcript once while listening to the audio recordings, the themes emerged from the lived experiences with IBS. From the themes, the essence of living with IBS was approached; 4- Describing the phenomenon through the art of writing and rewriting: the researcher wrote the extracted essential themes from the interviews as the story several times for a rich description of the lived experience of IBS patients, 5-Maintaining a strong and oriented relationship with the phenomenon: this phenomenon in this stage was maintained considering the question of “What is the lived experience of IBS patients?”; and, 6- Balancing the research context by considering the parts and the whole: the researcher maintained the relationship between the parts and the whole in her mind. 

In order to facilitate the analysis, we used MAX-Q version 7. The study of interviews as holistic resulted in creation of 17 descriptions from 17 interviews which had been carried out. Based on Van Mannen(20) viewpoint, in order to increase the criterion validity of the study, the interviews were guided with purposeful, original sampling in the direction of answering the main question of the research, i.e. “What is the meaning (nature) of living with IBS”? 

The researcher was in contact with the participants during the interview process for a long time. In order to ensure the trustworthiness of the study, Lincoln and Guba(21) criterion was used. For the credibility of the research, the results were presented to participants and they expressed the coordination of results with perception during life with IBS. To meet this criterion, the experts’ analysis and prolonged engagement were used. By doing some measures like member checking and experts’ analysis, the dependability of the study was ensured. By thick and vivid descriptions in the text, effort was made to prepare the background for judgment and evaluation of the others regarding the transferability of the results. Preserving the careful documentations at all stages of the research, and also efforts to obtain the comments of professors of the department of Persian literature, psychiatric nursing and gastroenterologists in this regard helped the confirmability of the research.

The Research Ethics Committee of Shiraz University of Medical Sciences approved this study to be conducted (Issue: IR.SUMS.REC.1394.S137). Ongoing informed consent was obtained from the participants after written and verbal explanations were provided as the patients’ consent was necessary for each stage of the research process. The participants were reassured about the confidentiality of the data. It was also emphasized that in case they no longer wished to participate or wanted to withdraw from the study at any time, there would be no effect on their treatment and care.

## Results

Participants included 15 patients with an age range of between 21 to 73 years and mean age of 37.53 years; they were 10 women and 5 men. The time range of affliction with IBS was 1.5 to 30 years with a mean of 7.8 years. In Table 1, demographic characteristics of the participants are shown. 

**Table 1:** Demographic characteristics of the participants in the study

	Sex	Age (years)	Marriage status	Education	Occupation	No. of children	Affection History (years)	Type of disorder
1	Male	35	Married	B.Sc.	Headmaster	2	15	Mixed
2	Female	30	Married	Diploma	House keeper	2	10	Mixed
3	Female	30	Married	M.Sc.	House keeper	2	10	Constipation
4	Male	39	Married	B.Sc.	Employee	1	8	Diarrhea
5	Female	41	Married	Associate degree	House keeper	0	5	Mixed
6	Male	27	Married	Under diploma	Worker	1	4	Diarrhea
7	Male	28	Single	Associate degree	Farmer	0	3	Mixed
8	Male	73	Single	Diploma	Retired nurse	0	30	Constipation
9	Female	42	Married	Under diploma	House keeper	1	3	Mixed
10	Female	36	Married	Under diploma	House keeper	1	6	Mixed
11	Female	40	Divorced	Under diploma	Employee	1	8	Constipation
12	Female	21	Single	Diploma	Tailor	0	2	Mixed
13	Female	36	Single	B.Sc.	House keeper	0	6	Diarrhea
14	Female	55	Widow	Diploma	House keeper	4	5	Constipation
15	Female	30	Married	B.Sc.	House keeper	1	1.5	Mixed

The appeared themes emerged from the data of this study were all around the lived experience of patients with IBS. These themes emerged from about 1500 thematic phrases, sentences or paragraphs from the interviews. Firstly, these phrases, sentences or paragraphs formed 8 themes and 25 sub-themes out of total interviews and then at a back and forth process and by merging these themes, lived experience of patients with IBS emerged in the form of 1 theme, 2 sub-themes and 5 sub-sub-themes. The emerged theme, relative sub-themes and sub-sub-themes of each one.

According to the descriptions of patients, the concept of living with IBS was the theme of “Transient and Crisis Liberation”. This theme has been formed from sub-themes of “storm in corporeality” and “relief”. In fact, patients with IBS experienced the “transient crisis and liberation” with feeling of storm in corporeality and relief. Participants in the current study faced the crisis in parts of their life, especially during the incidence of attacks of IBS. “Liberation” resembles the possibility of going beyond “current situation” to “other situation”. These patients experienced the feeling of relief and comfort in some days of their life or at the time of ending the attacks. They have somehow tackled with symptoms of IBS with fluctuations, as a tide. This theme has been linked with the meaning of living with IBS to such an extent that one of the participants, stated that *When I was in a wedding party, I felt uncomfortable because of signs of my illness and I just wanted to go as soon as possible and get free* (P.4). This exemplification associates the theme of Transient Crisis and Liberation in the mind of any reader excellently. 

### Storm in the Corporeality

One of the sub-themes of transient crisis and liberation was the concept of storm in the corporeality. Meaning and purpose of storm in the corporeality was the severe bodily symptoms like hard wind or heavy rain so that these symptoms were accompanied with tension and this tension led to repetition of the signs. These patients also spend moments with pain and severity resulted from the annoying and frustrating symptoms. For example, the descriptions of the following participant reflect the concept of storm in the corporeality in the mind of any reader clearly. A woman said in this regard with a garbled face that, *the similarity of living with syndrome….is like that, there is war and fracas in the body and earthquake is coming* (P.15). The experience of storm in the corporeality of patients with IBS meant “body in pain and affliction”, “tension and symptom sequence” and “distress in the moments of life”.

### Body in Pain and Affliction.

The statements mentioned from the participants that indicate the existence of resistant, intolerable, unpredictable and variable defecation signs plus comorbidity diseases with IBS illustrate the concept of “body in pain and affliction” Well. For example, a participant with a sullen face said that, by hearing the word IBS, its harassments come to his mind said that: *its harassment means you feel heavy and pain in your abdomen; it seems like stone, a disease whose pain is so resistant. In spite of the existence of so many physicians and treatment, it is inscrutable like a stone* (P.3). 

Many participants mentioned the painful defecation as frequent excretion immediately after seeing or eating food or even by hearing the name of food. It is necessary to mention that all the participants in their descriptions suggested the feeling of having no complete discharge after each time of defecation. This is an indication of defecation with persecution and facing patient’s body with pain and affliction. Participants variously mentioned the complications, symptoms or comorbidity with IBS which all indicate the cases like arthritis, depression, anxiety, intestinal prolapse and failure to control excretion, headache, sleep problems, bad smell of the mouth, sexual problems, fatigue, transpiration, faint position, falling down, and obsession. For example, a participant, regarding chronic fatigue syndrome stated that: *my body is weak; I get tired soon; I cannot do my works quickly and on time; and I get a headache. That’s all due to the syndrome* (P.1). Some of the propounded psychological aspects by participants played a role in the formation of the concept of body in pain and affliction. For example, one of the participants said in this regard that: *I cry with happy music, too* (P.12). Some of participants explained their experience about sexual problems resulting from IBS. It is clear that all conditions accompanied with IBS, either physical or psychical, that were described by the participants in different ways indicate the existence of pain and affliction in the body of the patients with IBS which is somehow associated with the storm in corporeality. Another sub-sub-theme forming the theme “transient crisis and liberation” during lived experience with IBS was “tension and symptom sequence” which is quite related to the “storm in corporeality” sub-theme.

### Tension and Symptom Sequence.

Tension and symptom sequence was one of the sub-sub-themes which played a role in the formation of storm in corporeality. They explicitly mentioned their lived experience and tension with IBS in their statements as the cause of symptoms of IBS and also propounded the relative symptoms as stressors. Before any dialogue, we point to a story from a participant that reflects the tension and symptom sequence properly. *I never forget when we went to Esfahan during my pregnancy. They had a playful child. I had no way. I sat in the toilet for a long time. I could not stand up. It was not finished. I wound up with pain. Their child was repeatedly saying that …Why does not my aunt come out. I was so embarrassed. That moment was painful and I became nervous. I cried. I was shedding tears involuntarily. This is a very bad feeling (tears were gathering in her eyes). I could not control the situation …* (P.3). Many participants mentioned in different ways the stressors of symptoms of IBS which were all indications of cases like nervous excitements resulting from family quarrels, hearing bad news in our journey, seeing the opposite sex during adolescence, occupational inconstancy of the spouse, and pregnancy. Most participants stated that as soon as they went out of house, the symptoms of IBS started. Some participants stated that they suffered from the attacks of IBS, 3 to 4 times per month and each time it lasted for two days. 

### Distress in the moments of life.

Participants in the study who emphasized their expressions on “body in pain” and “tension and symptom sequence”, experienced “distress in the moments of life too”. All these cases faced the patient with IBS with “storm in corporeality”. Participants pointed in different ways to the situations like having the possible worst situation at the time of fecal incontinency, feeling of explosion due to severe constipation and distension, displeasure from eating and moments of life, late elapsing of time in party and being harassed from the permanent mental engagement with IBS. All aforesaid cases played a considerable role in the formation of “distress in the moments of life”. The participants in the study pointed to the mentioned cases with various interpretations that all are indications of feeling of distress in the moments of life.

### Relief

It is estimated from descriptions of the participants that patients with IBS are faced with a problem in fulfilling their physiological basic requirement that is defecation. This fact encounters them with crisis. When this physiological requirement is fulfilled well, they have the feeling of freedom and liberation. According to the participants in the study, it could be said that the prettiness of days without IBS, enjoying the party and lack of attention to t time during privacy are related to the “moments with pleasure”. All aforesaid cases lead to relief in the individual with IBS. The second sub-theme related to “transient crisis and liberation” was relief. This sub-theme referred to the meaning of “feeling of release from bodily symptoms” and “moments with pleasure”.

### Feeling of release from bodily symptoms.

Patients mentioned the following cases about “feeling of release from bodily symptoms” in their experiences. Feeling calm and light was followed by complete evacuation of the digestive system, not being afraid of going everywhere and feeling of release and liberation at the time of lack of symptoms. A participant described the feeling of liberation as a flying butterfly at the time of lack of IBS: *Once I was looking at children’s program on TV. Children were playing on grass field, opened their hands and butterflies were moving above their head. I felt that I was feeling relief and liberation like these children at those times when I was not suffering from the syndrome* (P.10).

### Moments with pleasure.

Moments with pleasure were such experiences that confront the patient with IBS with comfort. As mentioned, moments with pleasure means beautiful days without the symptoms of IBS, enjoying the party and eating and lack of attention to time in our privacy. A participant stated that: *The days when I am not faced with the syndrome, it seems that I am living like other human beings and I enjoy. O’ God, the days that I do not have this problem are so beautiful* (P.5). 

Many participants described the sympathy of relatives when symptoms appear as the factor of improvement of the symptoms and having moments with pleasure. A participant mentioned that moments with pleasure occur while I do not realize the passage of time. At some moments like loneliness, being at journey or lack of disorder attacks, the time is passed in such a way that the patient does not pay attention to its passage and enjoys those moments. Some participants enjoyed drinking herbal tea and had a good feeling from drinking effective distillates on the symptoms of IBS. 

## Discussion

The main theme “Transient Crisis and Liberation” emerged in the present study. Actually, the meaning of living with IBS has been “Transient Crisis and Liberation”. Lämås *et al.*(22) showed in a study that Swedish middle-aged women described the lived experience with constipation under the title of “being alone at excruciating situation” in relation with “turbulence between feeling of torture and feeling of liberation and release” and “living with permanent tension and anxiety”. This result is similar to the findings of the present study. In the current study, the theme “Transient Crisis and Liberation” that includes two sub-themes of “storm in corporeality” and “relief” describes the attacking nature and unpredictability of living with IBS. 

The sub-theme “storm in corporeality” was related to the “body in pain”, “tension and symptom sequence” and “distress in life moments”. According to the statements of participants, this sub-theme expresses that sometimes the body of the patients with IBS suffers intolerable and resistant to treatment symptoms. They also sometimes face the same symptoms followed by the minimum tension whether physical, chemical, psycho-social, psycho-chemical and economical. Many of these patients suffer gradually from situations like chronic fatigue, arthritis, depression and anxiety, too. These cases are called co-morbidity diseases which all are indications of body in pain. All these cases cause tension in them and lead to the repetition of symptoms related to the disorder. Actually, tension and symptom sequence occurs. During these times, patients will not enjoy their moments of life and suffer from distress in those moments. 

The body is the base of our perception. During sickness, the individuals’ awareness of the world is disturbed and the body experiences the pain differently from what was expected. In one’s daily life and during health, the body is normal, but at the time of sickness, it loses its piece and cannot be normal and there exists a deep feeling of losing the bodily overall coherence.(23) In this regard, Schneider and Fletcher(13) in Canada illustrated the negative effect of IBS /Inflammatory Bowel Disease (IBD) on the physical and psychological dimensions of life of 7 women aged 18-22 years and found similar results. The experience of the mentioned women was in the form of anxiety reaction that resulted from the attack of IBS. This attack acted as a stimulator of the cascade of physical and psychological effects and they mentioned the permanent and intolerable pain as physical effects. Considering the fact that in the current study the body has been also in pain, the results of the above mentioned study was similar to those of the current study. In this regard, participants in a research carried out by Bertram *et al*.(24) also acknowledged the attacking and unpredictability of the symptoms.

The comparison of the results of the current study with those of the research conducted by Farndale and Roberts in which the experience of patients with IBS regarding the effect of disorder on their daily life and its psycho-social consequences has been studied seems to be logical. The reason for such comparison was to find out the emotional effects of living with IBS. Such effects are being created as emotional cycle beginning from the fear of inaccurate diagnosing of disorder in patients. Fear results from tension in them followed by worsening of the symptoms and, recreation of fear.(14) Generally, any type of tension could lead to activation of IBS,(25) but continual and chronic stressors are of more importance compared with acute stressor incidents.(26) Participants in the current study also declared that any type of tension causes appearance of symptoms of IBS and then the symptoms resulted in tension. Such forward-backward movements lead to tension and symptom sequence. Also, accompanying the courses of IBS with tension-in such a way that tension caused the symptoms and symptoms in turn caused the tension-was among the descriptions of Swedish patients with IBS in Jacobson *et al.*’s study.(27) This point is similar to the results of our study. On the other hand, the lived experience with constipation among Swedish middle-aged women was described by Lamas et-al. as being alone with ex a devastating situation causing physical and psychological tensions for them. The physical discomforts included feeling of sickness, having colic and inflation, and tolerating severe and painful defecation. In this study, living with permanent tension and anxiety was among the themes emerging from the descriptions of patients with constipation(22) that is similar to the results of our study, too. 

The participants of our study stated that they were suffering from the comorbidity of disorders with IBS like anxiety and depression which are two disorders related to tension,(28) so these disorders caused their body to be in pain. Other study also indicate the high repetition of these two disorders in patients with IBS.(29) Therefore, the effects of psychological aspect of this disorder should be considered by nurses and other health care providers. Participants in the current study described a part of the meaning of storm in corporeality as distress in moments of life. Jacobson *et al* .(27) studied the experiences of Swedish patients with long-term affliction with IBS in their daily life and showed that their experience was not definite cure and domination on IBS, but it was only improvement of the disorder. Patients were living with intermittent interaction between being good and illness. Researchers inferred the sub-sub-theme “tension and symptom sequence” from the descriptions of participants with phrases like feeling bad at moments of life which could be enjoyable;(27) this is similar to the results of the current study.

Two sub-sub themes of “feeling of release from bodily symptoms” and the “moments with pleasure” formed the “relief” sub-theme which includes the extensive meaning of descriptions of the participants regarding the under studied phenomenon. The perception of patients from “relief” not only included “feeling of release from bodily symptoms”, but also consisted of “moments with pleasure” in relation to the beauty of days without IBS, the enjoyment of using herbal medicines, enjoying the party, lack of attention to time during privacy and the sympathy of relatives for improvement of symptoms of IBS. In the study of Bengtsson *et al*.(25) the perception of Swedish patients with IBS from the good quality of life was related to sub-themes of not having anxiety and pain, sense of peace and not having tension, feeling of fitness and comfort and liberation from symptoms of IBS.(30) In a research by Lämås *et al.*,(22) middle-aged female participants with constipation experienced feeling of liberation and having the best days at the time of having bowel movements. Lived time from the view point of Van Mannen(20) is our temporary way in the world. 

Patients with IBS in the world with this disorder have had the feeling of comfort and liberation in the days without symptoms or at the moments of ending attacks. Patients of the current study described that in the toilet if the door is open before their entrance and also existence of good smell over there, they feel comfortable. Patients always wanted to be near the toilet and have private toilet so as to feel comfortable. These descriptions are similar to the results of the study of Faulkner(31) regarding the life of young adults with IBD. IBD consists of two Ulcerative Colitis and Crohn disease which occur due to organic reasons but produce digestive symptoms like IBS. However, “transient crisis and liberation” is a meaning of living with IBS that have been emphasized with similar terms both in this study as well as in the studies of developed countries. However, regarding the relative acceptance of views in other developing countries, the need for further studies is felt. There was no special limitation in this study. 

To the best of our knowledge, this study is the first qualitative research in this regard in Iran. It is suggested that the future studies survey the experiences of nurses, physicians and families of such patients so as to obtain deeper insight regarding IBS.(32) Researchers hope that the results of this study could be a right foundation for the functioning of treatment-health teams, especially nurses, and become effective in decision making and orientation of measures related to the care of such patients. Although lack of generalizability of the results of qualitative research is the characteristic of such studies, it may be considered as one of the limitations of this type of research from the view point of those who give great importance to practical use of the results of research.

## References

[1] Drossman DA, Morris CB, Schneck S, Hu YJB, Norton NJ, Norton WF (2009). International survey of patients with IBS: Symptom features and their severity, health status, treatments, and risk taking to achieve clinical benefit. Clin. Gastroenterol..

[2] Jahangiri P, Jazi MS, Keshteli AH, Sadeghpour S, Amini E, Adibi P. (2012). Irritable Bowel Syndrome in Iran: SEPAHAN Systematic Review No. 1. Int. J. Prev. Med..

[3] Ganji A, Malekzadeh F, Safavi M, Nassri-Moghaddam S, Nourie M, Sh Merat (2009). Digestive and liver disease statistics in Iran. Middle East J. Dig. Dis..

[4] Khademolhosseini F, Mehrabani D, Nejabat M, Beheshti M, Heydari ST, Mirahmadizadeh A (2011). Irritable bowel syndrome in adults over 35 years in Shiraz, southern Iran: prevalence and associated factors. J. Res. Med. Sci..

[5] Farzaneh N, Ghobakhlou M, Moghimi-Dehkordi B, Naderi N, Fadai F. (2013). Anxiety and depression in a sample of Iranian patients with irritable bowel syndrome. Iran J. Psychiatry Behav. Sci..

[6] Tamannaifar MR, Akhavan-Hejazi Z. (2013). Comparing the mental health and quality of life in patients with irritable bowel syndrome and healthy subjects in Kashan, Iran. Feyz.

[7] Roshandel D, Rezailashkajani M, Shafaee S, Zali MR. (2007). A cost analysis of functional bowel disorders in Iran. Int. J. Colorectal Dis..

[8] Moghimi-Dehkordi B, Vahedi M, Pourhoseingholi MA, Khoshkrood MB, Safaee A, Habibi M (2011). Economic burden attributable to functional bowel disorders in Iran: a cross-sectional population-based study. J. Dig. Dis..

[9] Lacy BE, Lee RD. (2005). Irritable bowel syndrome: a syndrome in evolution. J. Clin. Gastroenterol..

[10] Lackner JM, Mesmer C, Morley S, Dowzer C, Hamilton S. (2004). Psychological treatments for irritable bowel syndrome: a systematic review and meta-analysis. J. Consult. Clin. Psychol..

[11] Fawcett J, Desanto-Madeya S. (2013). Contemporary nursing knowledge: analysis and evaluation of nursing models and theories..

[12] Drossman DA, Chang L, Schneck S, Blackman C, Norton WF, Norton NJ. (2009). A focus group assessment of patient perspectives on irritable bowel syndrome and illness severity. Digest. Dis. Sci..

[13] Schneider MA, Fletcher PC. (2008). ‘I feel as if my IBS is keeping me hostage!’ Exploring the negative impact of irritable bowel syndrome (IBS) and inflammatory bowel disease (IBD) upon university‐aged women. Int. J. Nurs. Pract..

[14] Farndale R, Roberts L. (2011). Long-term impact of irritable bowel syndrome: a qualitative study. Prim. Health Care Res. Dev..

[15] Fletcher PC, Jamieson AE, Schneider MA, Harry RJ. (2008). "I know this is bad for me, but...": a qualitative investigation of women with irritable bowel syndrome and inflammatory bowel disease: part II. Clin. Nurse Spec..

[16] Fletcher PC, Schneider MA, Van Ravenswaay V, Leon Z. (2008). I am doing the best that I can!: Living with inflammatory bowel disease and/or irritable bowel syndrome (part II). Clin. Nurse Spec..

[17] McCormick JB, Hammer RR, Farrell RM, Geller G, James KM, Loftus EV (2012). Experiences of patients with chronic gastrointestinal conditions: in their own words. Health Qual. Life Outcomes..

[18] Håkanson C, Sahlberg‐Blom E, Nyhlin H, Ternestedt BM. (2009). Struggling with an unfamiliar and unreliable body: the experience of irritable bowel syndrome. J. Nurs. Healthcare Chronic Illn..

[19] Navab E, Hajibabaee F. (2016). Phenomenology with focusing on Van Manen methodology. Tehran University of Medical Sciences..

[20] van Mannen M. (2014). Phenomenology of practice. Meaning- Giving Methods in Phenomenological; Research and Writing..

[21] Lincoln YS, Guba EG. (1985). Naturalist enquiry..

[22] Lämås K, Anundsson E, Stare A-C, Jacobsson C. (2015). An interview study of the experiences of middle-aged women living with constipation. Clin. Nurs. Stud..

[23] Ohman M, Söderberg S, Lundman B. (2003). Hovering between suffering and enduring: the meaning of living with serious chronic illness. Qual. Health Res..

[24] Bertram S, Kurland M, Lydick E, Locke GR 3rd, Yawn BP. (2001). The patient's perspective of irritable bowel syndrome. J. Fam. Pract..

[25] Bengtsson M, Sjöberg K, Candamio M, Lerman A, Ohlsson B. (2013). Anxiety in close relationships is higher and self-esteem lower in patients with irritable bowel syndrome compared to patients with inflammatory bowel disease. Eur. J. Intern. Med..

[26] Bennett EJ, Tennant CC, Piesse C, Badcock CA, Kellow JE. (1998). Level of chronic life stress predicts clinical outcome in irritable bowel syndrome. Gut..

[27] Jakobsson Ung E, Ringstrom G, Sjövall H, Simrén M. (2013). How patients with long-term experience of living with irritable bowel syndrome manage illness in daily life: a qualitative study. Eur. J Gastroenterol. Hepatol..

[28] Carrasco GA, Van de Kar LD. (2003). Neuroendocrine pharmacology of stress. Eur. J. Pharmacol..

[29] Modabbernia MJ, Mansour-Ghanaei F, Imani A, Mirsafa-Moghaddam S A, Sedigh-Rahimabadi M, Yousefi-Mashhour M (2012). Anxiety-depressive disorders among irritable bowel syndrome patients in Guilan, Iran. BMC Res. Notes..

[30] Bengtsson M, Ohlsson B, Ulander K. (2007). Women with irritable bowel syndrome and their perception of a good quality of life. Gastroenterol. Nurs..

[31] Faulkner T-L E. (2008). Young adults living with inflammatory bowel disease - a phenomenological study..

[32] Mohebbi Z, Sharif F, Peyrovi H, Rakhshan M, Naini MA, Zarshenas L. (2017). Self-Perception of Iranian Patients during their life with Irritable Bowel Syndrome: A Qualitative Study. Electron Physician..

